# Atheroprotective Aspects of Heat Shock Proteins

**DOI:** 10.3390/ijms241411750

**Published:** 2023-07-21

**Authors:** Anastasia V. Poznyak, Varvara A. Orekhova, Vasily N. Sukhorukov, Victoria A. Khotina, Mikhail A. Popov, Alexander N. Orekhov

**Affiliations:** 1 Institute for Atherosclerosis Research, Osennyaya 4-1-207, 121609 Moscow, Russia; varvaraao@gmail.com (V.A.O.); vnsukhorukov@gmail.com (V.N.S.); 2 Institute of General Pathology and Pathophysiology, 8, Baltiyskaya St., 125315 Moscow, Russia; nafany905@gmail.com; 3 Department of Cardiac Surgery, Moscow Regional Research and Clinical Institute (MONIKI), 61/2, Shchepkin St., 129110 Moscow, Russia; popovcardio88@mail.ru

**Keywords:** atherosclerosis, heat shock protein, hsp, oxidative stress, cardiovascular disease

## Abstract

Atherosclerosis is a major global health problem. Being a harbinger of a large number of cardiovascular diseases, it ultimately leads to morbidity and mortality. At the same time, effective measures for the prevention and treatment of atherosclerosis have not been developed, to date. All available therapeutic options have a number of limitations. To understand the mechanisms behind the triggering and development of atherosclerosis, a deeper understanding of molecular interactions is needed. Heat shock proteins are important for the normal functioning of cells, actively helping cells adapt to gradual changes in the environment and survive in deadly conditions. Moreover, multiple HSP families play various roles in the progression of cardiovascular disorders. Some heat shock proteins have been shown to have antiatherosclerotic effects, while the role of others remains unclear. In this review, we considered certain aspects of the antiatherosclerotic activity of a number of heat shock proteins.

## 1. Atherosclerosis

The first inflammatory signs of atherosclerosis were described more than 150 years ago. However, interest in this topic has grown only over the past 30–40 years, during which innovative areas of fundamental scientific and clinical research have been actively developing. The majority of experimental and clinical reports on immunological phenomena in atherosclerosis were based on studies of progressive, clinically manifested lesions [1].

Atherosclerosis in both humans and animals is initiated by the infiltration of T cells into intima in classical predisposition sites, such as bends and arterial branches. The deposition of lipids and migration of macrophages and vascular smooth muscle cells is preceded by infiltration. It occurs when endothelial cells are stressed due to classic risk factors for atherosclerosis (hypertension, chronic infection, cigarette smoking, elevated lipid levels and diabetes mellitus) [2]. The consequence of this is an increased expression of heat shock protein (HSP)60. HSP is a group of proteins whose expression increases when cells are exposed to elevated temperatures or other stressors. Under non-stressful conditions, HSPs contribute to intracellular unfolding and folding, as well as to the transport and maintenance of proteins. The atherogenic effect of classical risk factors has been widely proven, but in this case they are considered as endothelial stressors at the earliest stages of atherosclerosis. Unlike venous cells, arterial endothelial cells have a lower threshold for stress effects. This is due to pre-stress, which is caused by a lifetime of higher blood pressure and different blood flow conditions in the arteries than in the veins [3].

HSP60 has a high degree of structural integrity and has existed for 2.5 billion years. Sequence homology is >95% between different bacterial species, and from prokaryotic to eukaryotic (including human) cells. HSP60 still has ~55% of total homology at the protein and DNA level, while in certain molecular domains this homology even exceeds 70% [4]. Cellular and humoral immunity against bacterial HSP60 in humans is produced as a result of vaccination or infection. At the same time, this protective reaction increases the risk of a cross-reaction with autologous HSP60, which is expressed by stressed arterial endothelial cells. Also, the immune system produces a true physiological autoimmune response against the biochemically modified autologous HSP60, which is expressed by damaged and dying cells and released from them (vascular endothelial cells) [5]. Similar biochemical modifications of HSP60 can be established thanks to Western blotting, due to the fact that the study of human HSP in the sera of patients with atherosclerosis leads to the formation of multiple protein bonds or smears [6].

So, if we consider atherosclerosis from an evolutionary point of view, it is a consequence of the impact of risk factors on the arteries. At the same time, modern risk factors emerged at a time when cultural evolution was ahead of biological evolution. Such consequences of modern life as smoking, diabetes, overweight and hypertension arose because during evolution these risk factors were not identified as selective pressure factors [7].

The concept of atherosclerosis as an autoimmune disease was first introduced in 1992. This article summarizes the early data on this theory for HSP60 and other HSPs, and also presents the results of recent research and describes promising developments in this area [8,9].

## 2. Cardioprotective Mechanisms of Hsp90

A common complication in diabetes is damage to the microvessels of the heart. It occurs with an increase in OS and inflammatory reactions and the induction of an unfolded protein response (UPR). In this case, UPR is an adaptive response mechanism. Excessive activation of UPR triggers a cascade of pathological events that lead to apoptosis in specialized tissues and cells. Sensitive inositol-requiring enzyme 1 alpha (IRE1a) plays a crucial role in diabetes-mediated microvascular damage [10]. This is due to the splicing of X-Box-binding protein 1 (XBP1), which further accelerates the degradation of intracellular vascular endothelial growth factor A (VEGF) and, due to this, interrupts vascular regeneration. The stability of IRE1a is determined by association with the cytosolic chaperone HSP90. Thus, UPR, the HSP90-IRE1a complex and endothelial dysfunction bind. Also, hyperglycemia caused by diabetes affects the glycation of proteins and lipids (end products of advanced glycation (AGEs)), which cause UPR and ER (endoplasmic reticulum) stress with further induction of apoptosis and deterioration of the integrity and function of the endothelium [11]. In addition, the protective role of cardiomyocytes against damage mediated by high glucose has been shown for both HSP90 (via the Akt pathway) and TRAP1 (TNF Receptor Associated Protein 1) through regulation of mitochondrial membrane potential (MMP), opening of the mitochondrial permeability transition pore (MPTP) and ROS (reactive oxygen species) levels [12].

**Figure 1:**
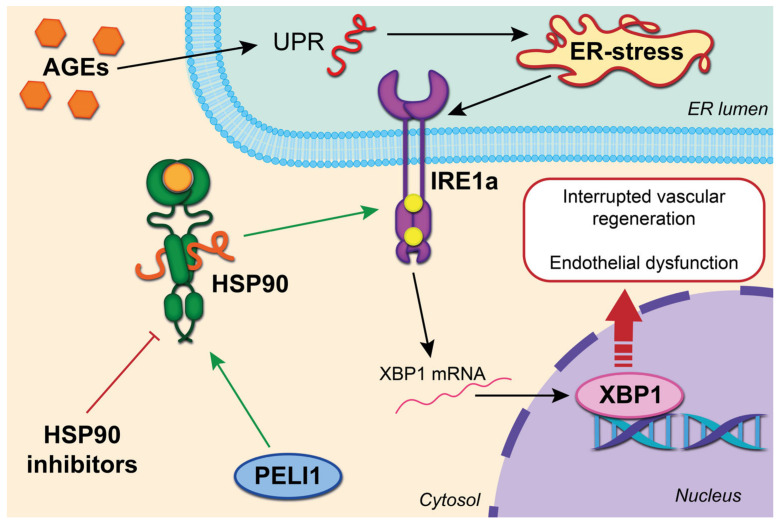
Schematic representation of effects of the HSP90 inhibitors.

An additional regulatory pathway was identified on the culture of microvascular endothelial cells of the heart of a mouse model with a deficiency of ubiquitin–protein ligase 1 Pellino E3 (PELI1), which is known as a stress mediator ER and stability regulator IRE1a. The ring domain of Peli 1 binds Hsp90, and also enhances the phosphorylation of IRE1a [13]. Thus, by limiting ER stress, preventing or blocking the interaction of Peli1 with HSP90 can protect against diabetes-mediated damage to the microvessels of the heart [14]. We summarize this process in Figure 1.

The ring domain of Peli 1 binds Hsp90, and also enhances the phosphorylation of IRE1. AGEs correspond to the UPR and ER with further induction of apoptosis and deterioration of the integrity and function of the endothelium. Splicing of X-Box-binding protein 1 (XBP1) further accelerates the degradation of intracellular vascular endothelial growth factor A (VEGF) and, due to this, interrupts vascular regeneration.

Recent studies have demonstrated that the 17-AAG HSP90 inhibitor (tanespimycin) inhibits ER stress and activation of nuclear factor kappa B (NF-κB) in rat cardiomyocytes. It was due to this that apoptosis was prevented. The action of 17-AAG is based on the normal expression of miR-93, which is an important regulator of cardiomyocyte survival under stress [15]. Similarly, a decrease in the rate of apoptosis of myocardial cells was observed on a model system of broiler chickens. The effect of the administered aspirin provoked increased regulation of HSP90 and AKT expression, and inhibition of the activity of caspase-3 and caspase-9 [16]. This was confirmed by the results of additional studies on a model of cardiac microvascular endothelial cells (CMVECs) affected by Hsp90 in rats. In these experiments, aspirin treatment stimulated the expression of Hsp90, which acts via Akt and PKM2 (pyruvate kinase 2/3) signals to protect CMVECs from heat stress damage [17]. Experiments on myocardial tissues of mice exposed to heat stress also showed that the administration of aspirin induces Hsp90 and Akt, stimulates PKM2 signaling, and accelerates mitochondrial translocation of Akt and Pkm2, which leads to phosphorylation of the mitochondrial regulator of apoptosis Bcl-2 (B-cell lymphoma 2) to protect the integrity of mitochondria from the initiation of apoptosis [18].

## 3. HSP60-Based Vaccination

Based on the results of a number of past studies conducted on rabbits, a mouse model of atherosclerosis has been developed, which is controlled by HSP60 and serves as a tool for HSP60-based vaccination [19]. The autoimmune concept of atherosclerosis underlies the idea that the development of tolerance to HSP60 and its peptides can be an effective solution in the prevention and treatment of atherosclerosis [20,21]. This principle has been successfully tested on various animal models. Thus, in mice with the low-density lipoprotein receptor knockout (LDLR^−/−^) with induced mycobacterial tolerance to HSP65, a significantly reduced lesion size was revealed, in particular the reduced reactivity of lymphoid cells to HSP60 and increased production of IL-4 [22]. These results support the idea that IL-4 (interleukin 4) plays a protective role during the development of atherosclerosis. There is also evidence that IL-4 can have both pro-inflammatory and anti-inflammatory effects on various parts of the vessels. Thus, the total effect of this cytokine may depend on the stage of the disease [23].

**Figure 2:**
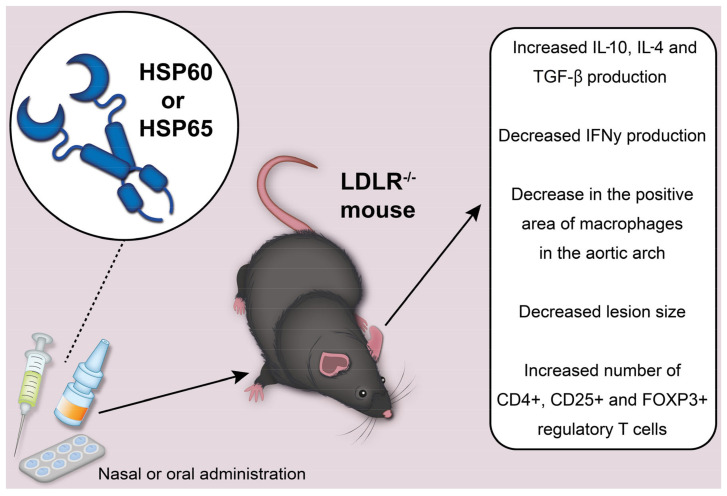
Effects of the use of HSP60 or HSP65 in LDLR^−/−^ mice.

Nasal and oral administration of Mycobacterium HSP65 to induce tolerance was studied in mice with the LDLR^−/−^. We depict the potential effects of HSP60 or HSP65 administration to mice in Figure 2. In mice injected with HSP65 nasally, there was a decrease in the positive area of macrophages in the aortic arch, a significant decrease in the size of the lesion, an increase in IL-10 production, a decrease in the number of CD4+ T cells and a decrease in IFNγ (interferon gamma) production [24]. Mice that received HSP65 orally showed a similar trend. At the same time, the differences with the control were not significant, except for the size of the lesion. In addition, reduced levels of antibodies to HSP60 were found in the group where nasal treatment was used. The immune response was attributed to type 2 T helper cells. This was reflected in significantly elevated IgG antibody titers, which corresponded to the cytokine profile found in these mice [25,26].

In another method of oral immunization against mycobacteria HSP65, genetically modified recombinant strains of Lactococcus lactis were used. They were used to deliver protein to the mucosa and induce intracellular or extracellular protein production. Due to this method, it was possible to weaken atherosclerosis in mice with the LDL^−/−^ receptor. Perhaps such antigen-specific tolerance was a consequence of the transition from the immune response of type 1 T helper cells to the response of type 2 T helper cells, as IL-10 concentrations increased and IFNγ levels decreased in vitro [27].

Oral tolerance to mycobacterial HSP65, its peptide and HSP70 peptide with common immunoregulatory properties was also studied in mice with the LDL^−/−^ receptor. In mice that received mycobacterial HSP65 and its peptide, a significantly reduced lesion size was observed in the carotid arteries and aortic root. In addition, after induced tolerance, the production of IL-10 and transforming growth factor β (TGF-β) increased [28]. The decrease in lesion size correlated with an increase in the number of CD4+, CD25+ and FOXP3+ regulatory T cells in several organs and with increased expression of Foxp3, CD25 and CTLA4 mRNA in lymphoid cells within atherosclerotic lesions. In one of the other studies, orally induced tolerance to a combination of apolipoprotein B peptide and HSP60 peptide prevented the progression of atherosclerotic lesions. It also provided plaque stabilization, the induction of CD4+, CTLA4+, CD4+, CD25+ and FOXP3+ regulatory T cells, and increased production of TGF-β [29,30].

Tolerance to HSP60 in mice, which was induced nasally, also led to the suppression of atherosclerosis, accompanied by a significant increase in CD4+, LAP+, CD4+, CD25+ and FOXP3+ regulatory T cells and a simultaneous increase in the production of TGF-β. It is also important to note that the injection of an antibody to TGF-β neutralized the protective effect of mycobacterium HSP65 [31]. Nasal administration of Mycobacterium HSP65 has also been associated with beneficial effects in rabbits (suppression of T cell proliferation, reduction of aortic lesion size, increased IL-10 production and absence of related antibodies). At the same time, the level of lipids in the blood serum was reduced. Thus, it can be said that an effective approach to prevent and mitigate atherosclerosis is to promote HSP60-specific tolerance by introducing HSP60 or mycobacterium HSP65 through the mucous membrane [32].

## 4. Endogenous HSP27 Overexpression Attenuates Atherogenesis and Inflammation

To study the functional significance of HSP27 in atherogenesis, both the early and late phases of lesion development were considered, using the example of a mouse model of inflammatory atherogenesis. Apoe^−/−^ mice were fed a high-fat diet (HFD). In order to determine whether HSP27 would change atherogenesis in this model, mice overexpressing human HSP27 (HSP27o/e) were crossed with Apoe^−/−^ mice [33]. According to the results of four weeks of HFD, women (but not men) had a 35% decrease in atherosclerotic load on the aorta Apoe^−/−^ HSP27o/e versus Apoe^−/−^ control mice. Despite the fact that the initial levels of HSP27 in the blood were almost imperceptible in all mice, at the end of 4 weeks of high-fat diet (HFD), female Apoe^−/−^ HSP27o/e mice had an increase in the level of HSP27 in the blood of more than 10 times (with a slight change in levels in male mice) [34]. There was also an inverse correlation between the level of HSP27 in the blood and the degree of atherosclerotic load. Treatment of rHSP27 macrophages in vitro was associated with a decrease in the formation of foam cells. This was reflected in a decrease in the absorption of acetylated LDL (AcLDL). These macrophages released less IL-1β (proinflammatory) and more IL-10 (anti-inflammatory) into the cell culture medium. Accordingly, it can be assumed that HSP27 indirectly influenced the production of inflammatory cytokines. Perhaps this was due to a decrease in the transformation of macrophages into foam cells [35].

**Figure 3:**
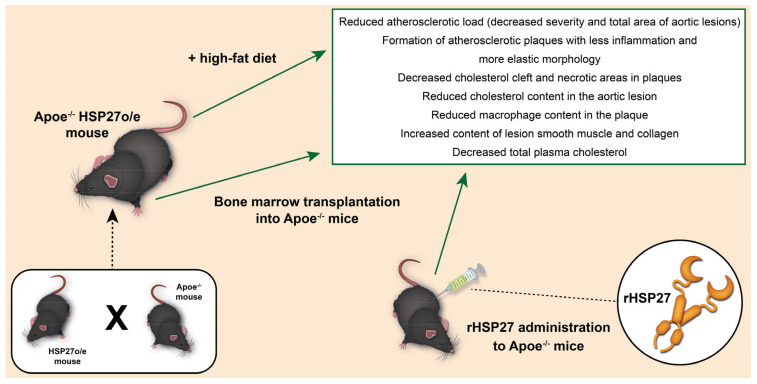
Changes in the morphology of the lesion in response to rHSP27 administration.

Since chronic overexpression of HSP27 promotes favorable plaque remodeling, it is atheroprotective in Apoe^−/−^ mice. In Apoe^−/−^ HSP27o/e mice fed HFD for 12 weeks, blood levels of HSP27 increased by more than 16 times compared to the initial values. It is important to note that blood levels of HSP27 were higher in female mice [33]. This is consistent with previous in vitro and in vivo studies on the key role that estrogens play in stimulating the extracellular release of HSP27. It is possible that in male mice, the increase in the level of HSP27 in the blood was caused by prolonged exposure to metabolic stress, which was caused by atherogenic HFD [36]. The decrease in atherogenesis was modest in Apoe^−/−^ HSP27o/e mice compared to Apoe^−/−^ analog mice (for example, in both sexes, the reduction in the area of aortic lesion was 21–35% for facial and 24–30% for transverse sections of aortic sinus tissue), and there were impressive changes in the morphology of the lesion (see Figure 3), which correspond to less inflamed plaques, including:

1. Reduction of cholesterol in the aortic lesion, which is reflected by the number of cholesterol gaps in the intima, as well as areas occupied by lipids and free cholesterol;
2. Reduction in the content of macrophages in the plaque;
3. Increased content of lesion smooth muscle and collagen;
4. Increased stiffness of the damage (which is reflected in the stress–strain curves obtained during ex vivo mechanical stretching experiments).

Taken together, these histological changes are consistent with plaque inflammation, which does not weaken and may be in the process of resolution. In the course of further studies, it is important to establish the long-term effects of HSP27 on atherogenesis. At the moment, it is known that Apoe^−/−^ HSP27o/e mice fed a regular chow-chow diet for 32 weeks show a persistent decrease (−29%) in severity of aortic lesion with a concomitant (−26%) decrease in total plasma cholesterol compared to Apoe^−/−^ control mice. Based on this, it can be assumed that HSP27 may have a long-term atheroprotective effect [35].

## 5. Exogenous HSP27 Is Atheroprotective and Attenuates Arterial Inflammation

Since the secretion of HSP27 by macrophages in vitro was demonstrated, there was need to determine which cells stimulated an increase in the level of HSP27 in the blood in vivo, by bone marrow transplantation from Apoe^−/−^ HSP27o/e (HSP27 overexpression) mice into Apoe^−/−^ littermates who do not have the HSPB1 gene encoding HSP27 [37]. Despite the fact that serum levels of HSP27 were not detected in control mice, bone marrow transplantation from Apoe^−/−^ HSP27o/e mice resulted in a noticeable increase in HSP27 levels in the blood and a decrease in the severity of aortic lesion (for example, −50% for the face and −28% for the aortic sinus cross sections). Also, other beneficial effects were noted in the plaques of mice that received HSP27o/e bone marrow (for example, a decrease in cholesterol cleft and necrotic areas of the nucleus) [38]. Based on the results obtained during this experiment, it can be concluded that HSP27 obtained from blood-borne cells is sufficient to weaken the formation of atherosclerotic lesions de novo. It may also overshadow the functional importance of HSP27 expression by the artery wall (i.e., smooth muscles or endothelial cells).

Recombinant HSP27 (rHSP27) was also synthesized to study its therapeutic effects in the formation of de novo lesions. For 3 weeks, subcutaneous injections of rHSP27 (100 mcg) were administered twice a day. Due to this, the level of HSP27 in the blood increased, also reducing the total area of the aortic lesion (31% and 40% for the analysis of the aorta en face and the aortic sinus, respectively) [39]. It is important to note that during the treatment of rHS27 (*p* < 0.001), the level of total cholesterol in blood plasma was 42% lower. This was due to a decrease in the content of very-low-density lipoproteins and intermediate cholesterol/low-density subfractions. The mechanisms involved in lowering plasma cholesterol levels are an active area of research in O’Brien’s laboratory, due to important transcriptional effects on key mediators of cholesterol metabolism [40,41].

## 6. HSP27 Stabilizes Existing Plaques and Lowers Inflammation

Since clinical therapy in patients with an established disease often begins after a myocardial infarction or stroke, the ability of rHSP27 to modulate the progression and morphology of established atherosclerotic lesions was tested. To establish the “initial” state of atherosclerosis, Apoe^−/−^ mice were fed an atherogenic diet for 4 weeks [42]. Then, to simulate lipid management strategies, such as lifestyle changes and/or statin therapy, they were switched to a regular chow-chow diet. After the development of these initial lesions, rHSP27 (100 mcg) or the carrier was administered twice a day for 3 weeks. Initially, the levels of total cholesterol in blood plasma were the same in both groups. They decreased after switching to the chow-chow diet, but were 27% lower when treated with rHSP27 during euthanasia (*p* = 0.004) [43,44]. The area of the aortic lesion was modestly but significantly reduced in the treatment of rHSP27 compared to the control, and was comparable to that in the initial atherosclerotic condition. This was evidence that the progression of the lesion had been stopped. Also, rHSP27 therapy was associated with the formation of plaques in the intima with less inflammation and structurally more elastic morphology. At the same time, it remains unclear whether all the beneficial effects of HSP27 on the morphology of the lesion are directly related to the effect of this protein on the biology of macrophages [45]. There are other possible cellular effects that should be considered. This also includes the role of HSP27 in accelerating the reendothelialization of arterial lesions, which occurs due to increased regulation of the vascular endothelial growth factor.

## 7. HSP27 Modulation of Inflammatory Signaling

A decrease in the formation of atherosclerotic lesions, either with endogenous overexpression of HSP27 or with the introduction of exogenous rHSP27, occurred in the presence of a moderate decrease in plasma cholesterol levels. Despite the fact that the pathogenesis of atherosclerosis is caused by the dual effects of hypercholesterolemia and inflammation, the main emphasis was placed on how HSP27 can interact with the main transcriptional regulatory pathway NF-κB (nuclear factor kappa-light-chain-enhancer of activated B cells), which plays a key role in the modulation of inflammation [33]. NF-κB transcription factors regulate a huge number and variety of target genes, including those involved in cell proliferation, apoptosis, cell responses to stress, inflammation, and both innate and adaptive immune responses. Usually found in the cytoplasm as inactive dimers associated with AκΒ, NF-κB translocates into the nucleus as soon as IkB is phosphorylated by the superior IkB kinase complex (IKK), which leads to the transactivation of many target genes, including many gene programs that are associated with atherosclerosis [46].

Thus, further in vitro experiments were required to eliminate the potentially interfering hypolipidemic effect of HSP27 and the resulting indirect effect on inflammation. During the study of macrophages in tissue culture, it was found that rHSP27 activates the NF-κB pathway and alters the subsequent transcription. As a result, the regulation of key pro- and anti-inflammatory cytokine genes such as IL-6, GM-CSF, TNF or IL-10 increases or decreases, which is quite predictable [37]. Although it is often assumed that NF-κB plays a pro-inflammatory role, this may be an oversimplification. While the “pan-hematopoietic” knockout of NF-κB reduces experimental atherogenesis, the knockout of macrophage NF-κB actually worsens atherogenesis. Since macrophages produce anti-inflammatory factors (IL-10) in response to HSP27, activation of NF-κB in some cases is a critical factor in determining the balance of inflammation [47]. But it should be borne in mind that changes may occur over time, depending on the stage of the disease (for example, an early inflammatory stage of atherogenesis or a later progressive remodeling of plaque). There is a version that the functional response of macrophages is determined by interactions between triggered transcription factors and depends on the microenvironment and interdependent signaling cascades. Also, recent studies are aimed at studying the role of intracellular HSP27 in the NF-κB pathway [48]. Thanks to these, there will be enough information to better understand whether extracellular HSP27 has similar or different effects as an intercellular messenger.

## 8. HSP70′s Influence on Atherosclerosis and the Endothelium

Endothelial cells play a crucial role in maintaining vascular homeostasis. Vascular wall cells are constantly exposed to various forms of mechanical stress. These forms of stress include tension accompanying growth or muscle movement and cyclic pressure surges and circumferential tension due to blood pressure and pulsation, as well as wall shear stress due to blood flow [49,50]. These forms of stress can act as modulators that maintain homeostasis within the vessel wall. Thus, mechanical stress regulates the production of several vasoactive mediators, including NO, prostacyclin, endothelin and thromboxane A2, by endothelial cells in the vessel wall. At the same time, when the intensity of these stimuli exceeds the normal range, as well as when the biological activity of the endothelial cells themselves decreases for some reason, cardiovascular diseases (atherosclerosis and hypertension) may occur [51].

In their study, Luo et al. used an experimental system consisting of sheets of rat artery endothelial cells to analyze the mechanism by which endothelial cells resist various forms of physical stress. These mechanisms were the increased regulation of HSP expression and formation of stress fibers (SF) [51]. According to the results of the conducted studies, it can be said that the formation of fibers caused by stretching stress was caused by HSP70. SFs mainly consist of actin filaments that pass through the basal cytoplasm of the cell, and are connected at both ends to the membrane protein integrin through adhesion proteins. Integrins also bind specifically to extracellular matrix components (such as fibronectin). Due to this, they enhance cellular adhesion to the extracellular matrix [52]. SFs play a fundamental role in maintaining cellular or epithelial integrity, as endothelial cells respond to high shear stress or tension by forming SFs. Inhibition of the increased regulation of HSP70 by quercetin resulted in a significant suppression of stretch-induced SF formation in endothelial cells [51].

NO derived from endothelial NO synthase (eNOS) is evaluated as a possible protective factor against atherosclerosis. Accordingly, an increase in the expression of eNOS or NO production by pharmacological intervention can inhibit atherosclerosis. Perhaps NO has a protective effect in low concentrations, inhibiting the activation/adhesion of leukocytes and platelets and the proliferation of smooth muscle cells. As one of the causes of endothelial dysfunction, a violation of the production of NO by the endothelium can occur in the early stages of atherosclerosis before macroscopic damage manifests itself [53]. In their work, Panjwani et al. found how stimulation of mouse and human macrophages HSP96 and HSP70 led to the induction of induced NOS and NO production. The results of their research have expanded the role of these HSPs in innate immune responses to another powerful and highly conserved function of antigen-presenting cells (APC) [54].

In the presence of hypercholesterolemia, chronic overexpression of eNOS accelerates the development of atherosclerosis. Apparently, in mice, apoE-KO/eNOS-Tg. and this eNOS dysfunction played an important role in the progression of atherosclerosis [55].

The early events of the atherosclerotic process are the attraction of monocytes and T-lymphocytes to the vascular wall, and the activation of endothelial cells. In intima, monocytes differentiate into macrophages. These macrophages secrete pro-inflammatory cytokines, which in turn cause inflammation in the atherosclerotic plaque. High LDL cholesterol increases the risk of atherosclerosis. At the same time, cholesterol, which accumulates in macrophages, is mainly formed from modified forms of LDL—oxLDL [56].

oxLDL increases the expression of HSP70 in human endothelial cells and in smooth muscle cells. Based on this, it can be assumed that HSP70 represents cellular protection against oxLDL toxicity [57]. In their study, Zhu et al. found that oxLDL can trigger the expression of HSP70 (the induced form) only in non-confluent endothelial cells. Cell proliferation is responsible for this expression [58]. It has also been suggested that in vivo rapidly growing cells, such as cells of areas susceptible to atherosclerotic lesions, are more sensitive to oxLDL toxicity than dormant cells, and that increased expression of HSP70 may give proliferating cells an increased chance of survival. Confirmation of this observation may have therapeutic use in atherosclerosis [59,60].

An induced form of HSP70 was found in areas of the arterial wall that are subject to stress (hypertension, oxidative explosion). Similar circumstantial evidence also points to the protective role of HSP70.

Pirillo et al. found that overexpression of HSP70 failed to protect transfected cells from oxLDL cytotoxicity, but gave them a higher sensitivity. This effect is specific to Oxldl, and may be related to the ability of oxLDL to induce apoptosis [61,62].

## 9. Is HSP70 Concentration Low as a Result of Atherosclerosis or Is Low HSP70 Concentration the Primary Event Leading to Atherosclerosis?

HSP70 is a cytoprotective molecule. With its deficiency, the vulnerability of the cardiovascular tissue to the effects of the environment and physical stressors increases [63].

Despite the fact that HSP70 is an intracellular protein, in the presence of stress it can penetrate the cell membrane and can be detected in plasma. A decrease in the level of HSP70 may be an early indication of the development of atherosclerosis. It was found that the level of HSP 70 is noticeably lower in the skeletal muscles of people with type 2 diabetes. It also became known that HSP70 is moderately lower in non-diabetic identical twins with a diabetic co-twin [64].

Zhu et al. found that people with HSP70 levels below the median had twice the risk of CAD than those with a level above the median. At the same time, the severity of the disease (namely, the number of damaged vessels) was inversely related to the level of circulating HSP70 [58].

In endothelial dysfunction, as a result of a decrease in the release of NO into the bloodstream, HSP70 levels may be low. Basically, NO has an oxidizing effect, due to which the expression of HSP70 increases. Also, some studies suggest that blocking the release of NO reduces the expression of HSP. Thus, low levels of HSP70 in patients with progressive atherosclerosis may manifest as a result of primary endothelial dysfunction. At the same time, HSP70 does not induce NO production by macrophages, and is similarly capable of inducing NO production by endothelial cells [65].

Martin-Ventura et al. have revealed that low levels of HSP70 may be associated with its degradation as a result of proteolytic activity tolerated by hemorrhages present in the culprit plaques. This may reflect a balance between its secretion by healthy arteries and its degradation by atherosclerotic proteases [66].

Some chronic inflammatory diseases, as well as atherosclerosis, are associated with age-related changes. There are many factors that cause the aging immune system to lose control. Such factors include the fact that the inducibility of HSP gradually decreases with age. The expression of HSP in cells depends on the activity of so-called heat shock factors (HSF).

In elderly people, the function of these transcription elements in cells decreases. Accordingly, the main factor in the biology of aging may be a decrease in the stress resistance of cells as a result of inadequate regulation of HSP [67].

## 10. Conclusions

In our review, we considered a number of aspects of the functioning of heat shock proteins that are directly related to counteracting atherosclerosis. For example, Hsp27, according to a number of studies, stabilizes atherosclerotic plaques and also reduces the level of inflammation, due to interaction with the main transcriptional regulatory pathway NF-κB. This aspect can be used beneficially used in the development of antiatherosclerotic therapy. In the course of further studies, it is especially important to establish the long-term effects of HSP27 on atherogenesis.

With regard to Hsp70, there is a hypothesis that this protein reflects the protection of the cell from the toxic effects of modified LDL particles. Thus, Hsp70 can be considered as the potential target for oxidative stress decrease, which is serious contributor to atherogenesis. Hsp60 is potentially the basis for creating a vaccine against atherosclerosis. The range of studies showed the potential of Hsp60/Hsp65 to prevent, or at least decrease, the severity of atherosclerosis.

Despite the insufficient understanding of the processes underlying the antiatherosclerotic effects of heat shock proteins, it is obvious that their further study of these processes and intermolecular interactions can shed light on some options for the containment of atherosclerosis and its negative consequences. More in-depth studies evaluating the mechanisms of Hsp involvement in atherosclerosis are needed. There is a potentially beneficial linkage between their action and inflammation, oxidative stress, and other components of atherosclerosis pathogenesis. Also, data obtained from the investigation of other diseases, such as cancer, can improve our understanding of the implication for Hsp in atherogenesis.

## Data Availability

Not applicable.
